# Knowledge, attitude and practices on notifiable diseases among environmental health practitioners in the City of Johannesburg: A cross-sectional study

**DOI:** 10.4102/hsag.v27i0.1980

**Published:** 2022-12-07

**Authors:** Velisha Thompson, Joyce Shirinde, Thokozani P. Mbonane

**Affiliations:** 1 Department of Environmental Health, Faculty of Health Sciences, University of Johannesburg, Johannesburg, South Africa; 2 School of Health Systems and Public Health, Faculty of Health Sciences, University of Pretoria, Tshwane, South Africa

**Keywords:** knowledge, attitude, practices, notifiable diseases, surveillance, disease investigation, environmental health practitioners

## Abstract

**Background:**

Notifiable diseases, a public health challenge in low- and middle-income countries, require mandatory reporting and play a significant role in disease prevention and control. Environmental health practitioners are responsible for reporting and investigating notifiable diseases.

**Aim:**

The study aimed to assess the knowledge, attitude and practices on notifiable diseases among environmental health practitioners within a metropolitan municipality.

**Setting:**

The study was conducted in the seven regions of the City of Johannesburg in Gauteng province, South Africa.

**Methods:**

A cross-sectional and descriptive study was used. One hundred and thirty-five participants were randomly sampled. The data were collected using a semi-structured questionnaire and analysed using version 27 of the Statistical Package for the Social Sciences (SPSS) software. All ethical considerations such as permissions, ethical clearance and informed consent were observed throughout the study.

**Results:**

The majority of participants (*n* = 64; 47.4%) were aged between 30 and 39 years and had a BTech/Honours degree (*n* = 106; 78.5%). A total of 106 (78.5%) participants had received formal training, while 83.7% (*n* = 113) of the participants understood notifiable diseases. Years of experience had a significant negative correlation with the ‘need to report notifiable diseases’ (*r* = −0.193; *p* = 0.025).

**Conclusion:**

The results could facilitate a knowledge improvement programme that includes a structured training programme and standard operating procedures. The study results cannot be generalised to the whole country; hence, the recommendation of a national survey on similar phenomena should be considered.

**Contribution:**

The study findings could assist in improving the role of environmental health services in reporting and investigating notifiable diseases.

## Background

Reporting of notifiable diseases is part of the surveillance system used by health professionals to monitor and report diseases in time to ensure adequate diagnosis (Jajosky & Groseclose 2004). Surveillance of diseases and notification are vital components of preventing the spread of notifiable diseases (Yoo et al. 2009). However, inaccurate and delayed disease reporting has rendered the public health system insufficient and dysfunctional (Malakoane et al. 2020). In South Africa, the notification of diseases is based on the National Health Act and its Regulations (South Africa 2004).

Environmental health practitioners have a key role in the prompt and appropriate reporting and investigating of notifiable diseases (Mbonane & Naicker 2020). The recent outbreaks of severe acute respiratory syndrome (SARS) and severe acute respiratory syndrome coronavirus 2 (SARS‑CoV‑2) pandemics exemplify the need for effective and well-functioning disease surveillance systems (Ibrahim 2020). Even the World Health Organization (WHO) has repetitively called for implementing measures to improve the responses to disease outbreaks in each country on an ongoing basis (Piomelli 2002; WHO 2007). Countries should continuously review the performance of their surveillance systems (Kruk et al. 2018). Under-reported diseases obscure the burden and prevent accurate disease estimates (Gibbons et al. 2014). According to Prüss-Ustün and Neira (2016), approximately a quarter of the global disease burden is linked to environmental risk factors. It is estimated that 23% of all deaths may be linked to environmental factors (Prüss-Ustün & Neira 2016). Environmental health practitioners are actively involved in collecting environmental epidemiological data to monitor patterns of diseases and develop targeted interventions to control and prevent the occurrence of diseases (Lebelo & Van Wyk 2019b). Hence, it is important to evaluate the role of environmental health in the prevention of diseases.

The National Department of Health identified a deficiency in collecting health statistics, which was seen as fragmented (Maphumulo & Bhengu 2019). Previous literature shows that in low- and middle-income countries, environmental health services are disorganised with no uniformity (Lebelo & Van Wyk 2019b; Mbonane & Naicker 2020). The findings of the study by Lebelo and Van Wyk suggested that there were gaps in investigating infectious diseases during outbreaks in the Ekurhuleni Metropolitan Municipality among environmental health practitioners (Lebelo & Van Wyk 2019a). The probability exists that the same problem may arise among environmental health practitioners in implementing Regulations for notifiable diseases. According to anecdotal evidence and few studies in South Africa, environmental health practitioners’ practices when reporting and investigating notifiable diseases are inconsistent because of poor knowledge (Kgolane 2020; Lebelo & Van Wyk 2019b; Mbonane & Naicker 2020). Inconsistency in rendering environmental health services has been highlighted in the United States of America (Selman & Green 2008).

This study aimed to assess the knowledge, attitude and practices (KAP) of environmental health practitioners in the City of Johannesburg on the notifiable diseases surveillance system.

## Research methods and design

### Study design, site and population

**FIGURE 1:**
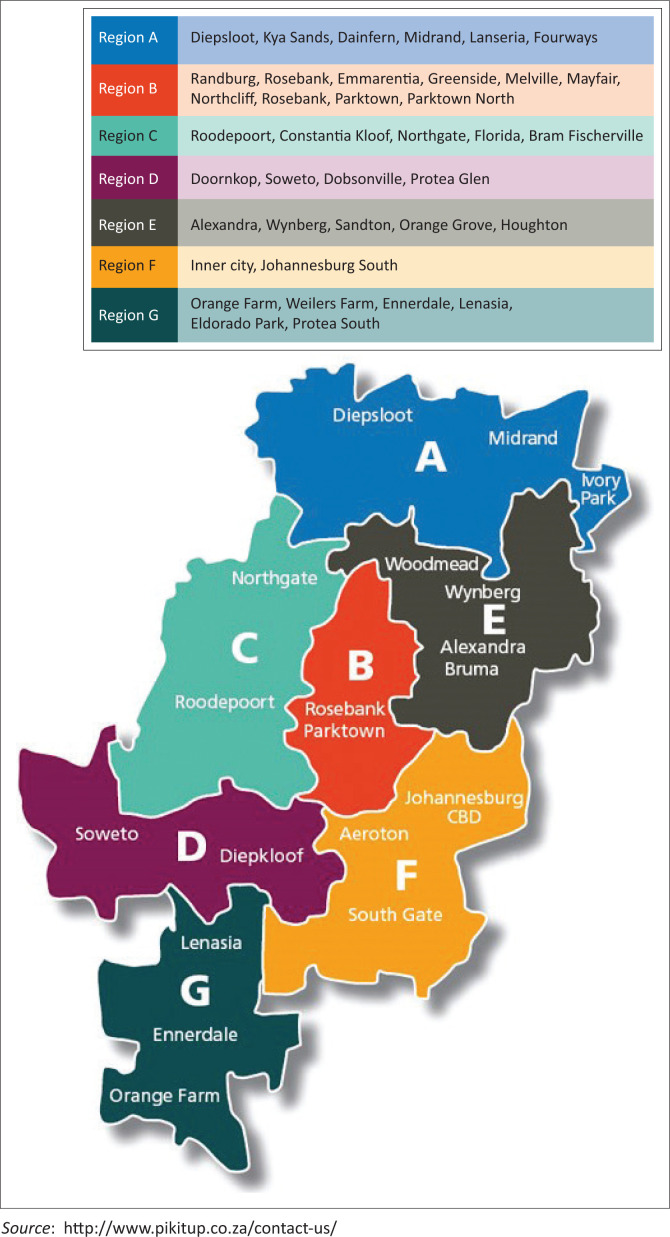
Map showing regions and main areas of the City of Johannesburg.

A descriptive cross-sectional study design was adopted and implemented to explore and assess the KAP among environmental health practitioners in the City of Johannesburg. The City of Johannesburg is South Africa’s biggest city situated in the province of Gauteng. The study population comprised all the environmental health practitioners (*n* = 209) employed in the City of Johannesburg, South Africa, within the city’s seven regions (Regions A to G), see Figure 1. The seven regions differ in terms of community services, for example, informal settlements, Central Business Area and suburb.

The sample size was determined using Epi Info version 7 (Centers for Disease Control and Prevention), based on the total estimated population of 209. To calculate the sample size, margin of error (ME) was set at 5%, confidence level was 95%, and the expected response rate was set at 50%. Therefore, the estimated sample size was 135.

The population was divided into seven clusters, namely Region A–G. Thereafter, participants were chosen using a simple random sampling within each cluster. The authors selected every second participant until they reached the required sample size. A response rate of 65% was achieved. The information and purpose of the study were explained to potential participants, individually.

### Data collection

Data were collected using an adjusted, self-administered and semi-structured questionnaire, which was adopted from previous studies that have studied other environmental health services (Lebelo & Van Wyk 2019a, 2019b; Mbonane & Naicker 2020). The researcher also considered current literature when developing the questionnaire. It was prepared and completed in English as the participants are conversant with the language because of their high qualification level. The questionnaire included sections on socio-demographics, knowledge, practices and perceptions of notifiable diseases. At the same time, the recommendations section contained open-ended questions. The questionnaire was piloted before the actual study. The completed questionnaires were either emailed or dropped in a sealed box placed in the secretary’s office of the Region Manager.

### Data management and analysis

Data were analysed using the latest version of Statistical Package for the Social Sciences (SPSS) (version 27) after processing (cleaning and sorting) in Microsoft Excel. The dependent variable for the study was practices, while socio-demographics, knowledge and perception were the independent variables. Descriptive analysis was used to show, summarise and describe quantitative data, while inferential analysis was conducted to determine any relationship between independent and dependent variables. Categorical variables were analysed and presented using frequency distributions in percentages and proportions. Pearson correlation coefficient was used to determine the relationship between dependent and independent variables. At the same time, Pearson’s chi-square test was used to determine statistical significance. Statistical significance was set at *p* < 0.05.

### Ethical considerations

The study obtained ethical clearance (REC-432-2020) from the Research Ethics Committee of the Faculty of Health Sciences, University of Johannesburg. At the same time, the Executive Director of the Department of Health (City of Johannesburg) permitted the researchers to approach and invite the environmental health practitioners to participate in the study. The environmental health practitioners gave informed consent prior to participating in the study.

## Results

A total of 135 environmental health practitioners participated in this study, as shown in Table 1. The majority of participants (*n* = 64; 47%) were in the 30–39 years age category, while 10% (*n* = 13) were older than 50 years. Most participants (*n* = 106; 79%) had a BTech/Honours degree qualification, followed by those with a National Diploma (*n* = 25; 19%). A few participants had a master’s degree (*n* = 2; 1%) or certificate/higher certificate (*n* = 2; 1%). There were 58 (43%) environmental health practitioners who had less than 5 years of experience in their profession; 24% (*n* = 33) had 6–10 years of experience, 20% (*n* = 27) had 11–15 years of experience, and 13% (*n* = 17) had 16 years or more years of experience. There were 106 (79%) participants who had received formal training in notifiable diseases, and 21% (*n* = 29) indicated no formal training in dealing with or managing notifiable diseases.

**TABLE 1:** Characteristics of participants (*N* = 135).

Characteristics	*N*	%
**Age (years)**		
20–29	41	30
30–39	64	47
40–49	17	13
50+	13	10
**Gender**		
Male	83	62
Female	52	38
**Qualification**		
National diploma	25	19
BTech/honours	106	79
Masters’ degree	2	1
Certificate/higher certificate	2	1
**Years of experience**		
0–5	58	43
6–10	33	24
11–15	27	20
16 or more	17	13
**Region**		
Region A	18	13
Region B	17	12
Region C	17	12
Region D	22	17
Region E	18	13
Region F	21	16
Region G	22	17
**Area of jurisdiction**		
Central business area	12	9
Informal settlement	34	25
Suburb	89	66
**Formal training on notifiable disease**		
Yes	106	79
No	29	21

Age had a negative significant correlation with ‘is there a need for notifiable diseases’ (*r* = −0.181; *p* = 0.035). Years of experience had a significant negative correlation with the ‘need to report notifiable diseases’ (*r* = −0.193; *p* = 0.025). Informal/formal training had a negative significant correlation with ‘source of information’ (*r* = −0.302; *p* = 0.000). Age had a negative significant correlation (*r* = −0.171; *p* = 0.047) with qualification. Gender (*r* = 0.341; *p* = 0.000) and age (*r* = 0.735; *p* = 0.000) had a positive significant relationship with the number of years of experience. Finally, there was a negative significant correlation between the number of years of experience and formal training received prior to taking part in the study (*r* = −0.265; *p* = 0.002).

### Knowledge and practices on notifiable disease surveillance and monitoring

There were 84% (*n* = 113) of environmental health practitioners who understood which diseases are not notifiable, and 16.2% (*n* = 22) did not know. Of the total study participants, 74% (*n* = 113) understood their role in dealing with notifiable diseases, whereas 16.3% (*n* = 22) were unclear. The majority of the participants (*n* = 113; 84%) understood the importance of collecting information on notifiable diseases. Most participants (*n* = 68) indicated that they had attended training and workshops on notifiable diseases that are provided by the City of Johannesburg, while 7% (*n* = 9) used sources from the library to get information on notifiable disease surveillance and monitoring. The majority of the participants (*n* = 90; 67%) reported having participated in an outbreak response within the last 6 months before taking part in the study, and 8% (*n* = 11) have never participated in outbreak surveillance or investigation. Of the total of 135 environmental health practitioners that took part in the study, 92 participants (68%) reported having collected data once a month from health facilities, whereas 28% (*n* = 38) claimed never to have collected any data from health facilities. The majority of the participants (*n* = 105; 78%) indicated using a standard operating procedure for notifiable diseases. The results show that many participants (*n* = 96; 71%) do not draw up action plans to address the disease burden in their respective areas of jurisdiction. Only 24% (*n* = 32) draw up action plans for disease burdens once a month. The relationship between socio-demographic and practice showed some correlation. Age had a weak positive correlation with ‘how often do you receive a disease notification’ (*r* = 0.185; *p* = 0.032). Qualification was negatively correlated with ‘how often do you receive a notification’ (*r* = −0.237; *p* = 0.006). Years of experience had a weak positive correlation with ‘what is the time frame for reporting’ (*r* = 0.224; *p* = 0.009). The study identified the following correlation between socio-demographics and knowledge: age had a negative significant correlation with ‘is there a need for notifiable diseases’ (*r* = −0.181, *p* = 0.035). Years of experience had a significant negative correlation with the ‘need to report notifiable diseases’ (*r* = −0.193; *p* = 0.025).

### Environmental health practitioners’ attitudes toward notifiable disease surveillance and monitoring

**FIGURE 2:**
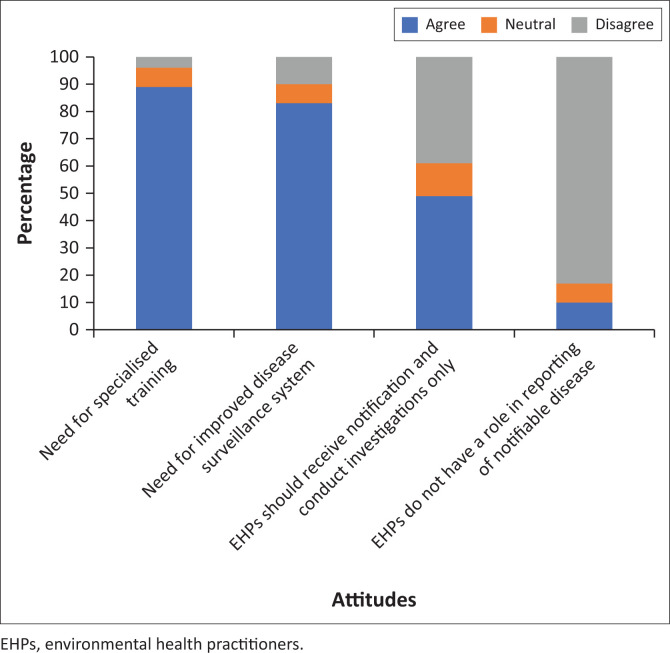
Attitude of environmental health practitioners on notifiable disease.

Figure 2 shows that 83% (*n* = 112) of the participants understood that environmental health practitioners are important in reporting notifiable diseases. Many participants, namely 49% (*n* = 66) agreed that environmental health practitioners are only responsible for receiving notifications and conducting investigations. Figure 2 also shows that 83% (*n* = 112) of the participants agreed that there is a need to improve the current disease surveillance system, whereas 10% (*n* = 13) of the participants disagreed. The majority of the participants agreed that there is a need for specialised training in disease surveillance. In contrast, a small percentage of participants (*n* = 5; 4%) disagreed about the need for specialised training in disease surveillance. Age had a significant positive correlation with ‘I believe environmental health practitioners do not have a role in reporting disease’ (*r* = 0.248; *p* = 0.004).

## Discussion

This cross-sectional study showed that most participants understood their role in the outbreak of diseases; however, there was a lack of knowledge in the description and characterisation of notifiable diseases. The knowledge and awareness of notifiable disease surveillance are key components for reporting diseases to the relevant authorities (Gauci et al. 2007). Health care workers with poor or inadequate knowledge may have missed or underreported cases of notifiable diseases (Mairosi et al. 2017). Only 47% of the participants (*n* = 64) could describe and characterise a notifiable disease in the current study. The current study results are similar to Mbonane and Naicker’s (2020) study, which found that environmental health had a knowledge gap in investigating communicable disease outbreaks. However, their study did not investigate or indicate the causes of the knowledge gap (Mbonane & Naicker 2020). According to other researchers, the knowledge gap can be attributed to the type of training a person was exposed to, the lack of capacity development on a particular function and the lack of specialisation in environmental health services (which equips an individual with specialised knowledge and skills) (Mbazima, Mbonane & Masekameni 2021).

Environmental health practitioners’ surveillance and prevention of communicable diseases are defined in the environmental health practitioners’ Scope of Practice of Environmental Health and are highlighted as an important role in preventing and limiting the spread of diseases (Agenbag & Balfour-Kaipa 2008). Most of the participants, that is 83% (*n* = 113), indicated that they understood their role in the surveillance and reporting of notifiable diseases. The results of this study are similar to the findings from a cross-sectional study conducted in Ekurhuleni Metropolitan Municipality, Gauteng province, by environmental health practitioners (Lebelo & Van Wyk 2019b). They found that environmental health practitioners knew their role in communicable disease surveillance in the Ekurhuleni Metropolitan Municipality.

The importance of training and development should be recognised as key elements for enhancing employees’ knowledge and skills in a work environment (Rodriguez & Walters 2017). In an information and technology-driven era, environmental health practitioners have access to numerous sources of information. The main sources of information the participants relied on were training and workshops arranged by the City of Johannesburg. Informal/formal training negatively correlated with ‘knowledge of standard operating procedures’ (*r* = −0.241; *p* = 0.005). Furthermore, there are numerous free virtual surveillance and outbreak investigation training and simulation-based e-learning platfoms available, internationally especially offered by the WHO and Centers for Disease Control. The City of Johannesburg can adopt this training as part of their training programme to allow environmental health practitioners to be exposed to real world scenarios and improve their knowledge and skills.

The significant negative correlation between training and understanding standard operating procedures may indicate that short and infrequent training sessions were available. A study by Kgolane (2020) found that environmental health practitioners were insufficiently trained to perform their activities effectively, in concordance with the findings of a study conducted in the Ekurhuleni Municipality that highlighted inadequate formalities training among environmental health practitioners on a specific function (Kgolane 2020; Mbonane 2015). Nevertheless, environmental health practitioners are expected to be continuously trained in keeping with trends relating to disease surveillance (Lebelo & Van Wyk 2019b). The study findings suggest changing practices of environmental health practitioners concerning disease surveillance, participation in outbreaks and reporting systems. Ninety (68%) of the participants reported participating in an outbreak response less than 6 months prior to the study. The balance reported that 12 months or more had passed since they last participated in an outbreak response. This could indicate that environmental health practitioners only become involved in an outbreak notification if it occurs in their respective area of jurisdiction.

Health care workers linked to the prevention and control of diseases must continuously collect, analyse and disseminate health-related data to inform preventative and control health interventions (Soucie 2012). Environmental health practitioners play an important role in preventing and controlling diseases. However, the study showed that only 92 participants (68%) reported collecting data once a month from health facilities, whereas 38 participants (28%) claimed never to have collected any data from health facilities. The routine data collection practices are concerning in the study especially considering that many participants have never conducted routine data collection on notifiable disease before. Environmental health practitioners are required by law to play a role in the surveillance and control of communicable diseases. Routine data collection from health facilities can assist in detecting ill health conditions affecting a particular community.

Lebelo and Van Wyk (2019b) revealed that standard operating procedures for environmental health are not fully implemented by environmental health practitioners, as they rely on passive surveillance of diseases and wait for disease notifications. A similar finding can be seen in this study, where environmental health practitioners are not fully conversant with timeframes for disease reporting. Only 22% (*n* = 30) of the participants reported no standard operating procedure for notifiable diseases, indicating a gap in the knowledge of time frames and standard operating procedures for diseases. There was inconsistency in the practices among participants within the different regions in the City of Johannesburg. These findings were similar to the Ekurhuleni Metropolitan Municipality among environmental health practitioners (Mbonane & Naicker 2020).

Disease notification systems are reliable and efficient providers for planning evidence-based interventions for public health trends and infectious disease outbreaks (Gibbons et al. 2014). Musoke et al. (2016), in their study, also concluded that environmental health practitioners have an important role in disease surveillance, its prevention and control, which therefore makes it necessary for plans to be devised to prevent the burden of communicable diseases (Musoke et al. 2016). However, this study shows that many participants (*n* = 96; 71%) do not draw up action plans to address the disease burden in their respective areas of jurisdiction.

In the current study, 112 (83%) participants had a positive attitude towards notifiable disease surveillance. Furthermore, they understood the important role in reporting notifiable diseases, and 89% (*n* = 120) of them agreed that there is a need for specialised training on disease surveillance. A similar finding was seen in the study by Lebelo and Van Wyk (2019b), which found that most of the environmental health practitioners in the Ekurhuleni Metropolitan Municipality knew that they have a significant role in active disease surveillance.

This study reported that most participants had a positive attitude towards notifiable disease surveillance and established a need for specialised training on disease surveillance. There was a positive correlation in the study between age and responsibility for receiving disease notifications and conducting investigations, indicating that the older environmental health practitioners have a more positive attitude towards disease surveillance and taking on more responsibilities.

This descriptive research study provided an in-depth view of the environmental health practitioners’ KAP regarding notifiable disease surveillance and reporting systems.

### Study limitations

The limitation of the study is that the study cannot be generalised to other parts of the province, South Africa and elsewhere because of different approaches to notifiable disease surveillance and monitoring. Hence, a national survey on a similar topic can describe and determine if the challenges identified in the current study are similar to other municipalities in the country.

### Recommendations

#### Knowledge management and improvement

Knowledge management refers to managing knowledge and information by developing, organising and sharing material within an organisation (Girard & Girard 2015). This can benefit the environmental health practitioners in the City of Johannesburg. A multi-disciplinary approach involving key stakeholders can systematically disseminate essential and pertinent information to environmental health practitioners through a technological medium. This holistic approach will allow the organisation to meet its goals and objectives and enable environmental health practitioners to make informed decisions on notifiable disease surveillance and reporting. There is a need to regularly evaluate the notifiable disease surveillance and reporting systems and their implementation to ensure quality assurance. This strategic approach will improve the inconsistencies and irregularities identified in this study within the different regions and environmental health practitioners.

#### Training and development

Many participants identified inadequate and sporadic training as a concern, and the need for more training was suggested. Well-structured and continuous training develops, empowers and motivates staff and improves their performance. Environmental health practitioners may benefit from training and development programmes by fostering staff engagement and communication to meet specified standards. Reinforcement of continuous training should include theoretical and practical work, group activities and assignments followed by an assessment that is linked to continuous professional development points. The training should be in line with Health Professions Council of South Africa (HPCSA) regulations relating to the registration by environmental health officers of additional qualifications. In addition, coordinated orientation programmes for newly employed environmental health practitioners with scheduled follow-ups should be conducted to ensure consistency with legislation, policies and procedures. Comprehensive, coordinated training sessions will improve the knowledge gap of environmental health practitioners identified in the study.

#### Development of standard operating procedure and guidelines

As Lebelo and Van Wyk (2019b) indicated, the lack of guidelines and standard operating procedures for communicable diseases could be a prevalent problem in the whole country. This study showed that in the City of Johannesburg, there might not be a documented standard operating procedure for environmental health practitioners on notifiable disease surveillance and reporting. A standard operating procedure should be developed in line with Norms and Standards requirements, the National Institute for Communicable Diseases of South Africa standard operating procedures, and the monitoring tool requirements of the City of Johannesburg. Standard operating procedures should be formulated together with environmental health practitioners, management and other key role players to allow for proper implementation by all environmental health practitioners. The standard operating procedure and guidelines will ensure consistency and uniformity in the practices of environmental health practitioners in the various regions. The standard operating procedure and guidelines should guide the environmental health practitioners on routine surveillance on communicable diseases, including data collection and analysis which should improve challenging practices.

## Conclusion

The study findings indicate that environmental health practitioners are aware of their role in communicable disease control. Yet, the lack of knowledge about the notifiable disease and its management system is a concern. The results implied that there might be a lack of uniformity and consistency in the knowledge and practices on notifiable disease surveillance among environmental health practitioners working in the different regions in the City of Johannesburg. However, the participants noted a need to improve the current disease surveillance system.
